# Five vs. two initial rescue breaths during infant basic life support: A manikin study using bag-mask-ventilation

**DOI:** 10.3389/fped.2022.1067971

**Published:** 2022-12-07

**Authors:** Anke Geerts, Sandrine Herbelet, Gautier Borremans, Marc Coppens, Erik Christiaens-Leysen, Patrick Van de Voorde

**Affiliations:** ^1^ Department of Basic and Applied Medical Sciences (BAMS), Ghent University, Ghent University Hospital, Ghent, Belgium; ^2^ Department of Emergency Medicine, Ghent University Hospital, Ghent, Belgium; ^3^ Federal Department of Health, EMS Dispatch Centre 112 Flanders, Ghent, Belgium

**Keywords:** infant CPR, manikin study, initial rescue breaths, bag-mask ventilation, pediatric, BLS

## Abstract

**Background and objectives:**

Children are more likely to suffer a hypoxic-ischaemic cause for cardiac arrest. Early ventilation may provide an advantage in outcome during paediatric cardiopulmonary resuscitation [CPR]. European Resuscitation Council guidelines recommend five initial rescue breaths [IRB] in infants, stemming from the hypothesis that rescuers might need 5 attempts in order to deliver 2 effective ventilations. This study aimed to verify this hypothesis.

**Methods:**

Participants (*n* = 112, convenience sample) were medical students from the Faculty of Medicine and Health Sciences Ghent University, Belgium. Students were divided into duos and received a 15 min just-in-time training regarding the full CPR-cycle using BMV. Participants then performed five cycles of 2-person CPR. The IRB were given by 1-person BMV, as opposed to a 2-persons technique during the further CPR-cycle. Correct ventilations for the infant were defined as tidal volumes measured (Laerdal® Q-CPR) between 20 and 60 ml, with *n* = 94 participants included in the analysis. The primary outcome consisted of the difference in the % of medical student duos providing at least 2 effective IRB between 2 and 5 attempts.

**Results:**

Off all duos, 55,3% provided correct volumes during their first 2 initial ventilations. An increase up to 72,4% was noticed when allowing 5 ventilations. The proportional difference between 2 and 5 IRB allowed was thus significant [17,0%, 95% confidence interval (5.4; 28.0)].

**Conclusion:**

In this manikin study, 5 IRB attempts during infant CPR with BMV increased the success rate in delivering 2 effective ventilations. Besides, students received training emphasizing the need for 5 initial rescue breaths. This study provides evidence supporting European Resuscitation Council guidelines.

## Introduction

Cardiac arrest [CA] is rare in children. Yearly, approximately 16.000 children worldwide suffer an out-of-hospital CA, 40%–50% of which are infants (1–4). Similar numbers are reported in paediatric in-hospital CA, averaging as low as 0.77 per 1,000 hospital admissions (2, 5). Although primary arrhythmias causing CA occur in children, most reported causes are hypoxic-ischaemic in nature (e.g., sudden infant death syndrome, respiratory infection, airway obstruction, hypovolemia …) (1, 4). The outcome of such CA is bad, and especially in infants only a small number survive with good neurological outcome (6–8).

In view of the above, despite limited evidence, the 2021 paediatric life support European Resuscitation Council [ERC] guidelines recognise the importance of timely ventilation (and oxygenation) as part of the paediatric basic life support [BLS] algorithm (1). Importantly, the authors advocate starting the sequence with five initial rescue breaths [IRB]. In early guidelines this used to be just two breaths (as in the further sequence) (9). Later paediatric cardiopulmonary resuscitation [CPR] guidelines recommend five attempts to at least deliver two effective ventilations. The fact the authors changed this in 2015 to five initial breaths regardless was driven by the yet unproven hypothesis that one initially needs more attempts to be effective (9).

With this manikin study, this hypothesis was assessed in the specific situation of paediatric basic life support [BLS] providers using bag-mask ventilation [BMV]. Although this question is equally relevant when applying mouth-to-mouth (nose) ventilation, due to the ongoing COVID pandemic this could not be evaluated.

## Material and methods

This study was approved by the ethics committee of the Ghent University Hospital. Medical students were recruited from Ghent University, Belgium, Faculty of Medicine and Health Sciences by posting a message on the official communication forum of the University (Ufora). Inclusion criteria were as follow: (1) being a medical student. (2) being able to perform 5 cycles of CPR. Exclusion criteria were defined as being an officially certified BLS provider or a lifeguard. Students already received proper BLS training during their regular medical curriculum but had limited to no experience with BMV. Given the near lack of proper *a priori* data and the anticipated recruitment difficulties within the available time period, we recruited a convenience sample of eventually 112 participants.

**Figure 1:**
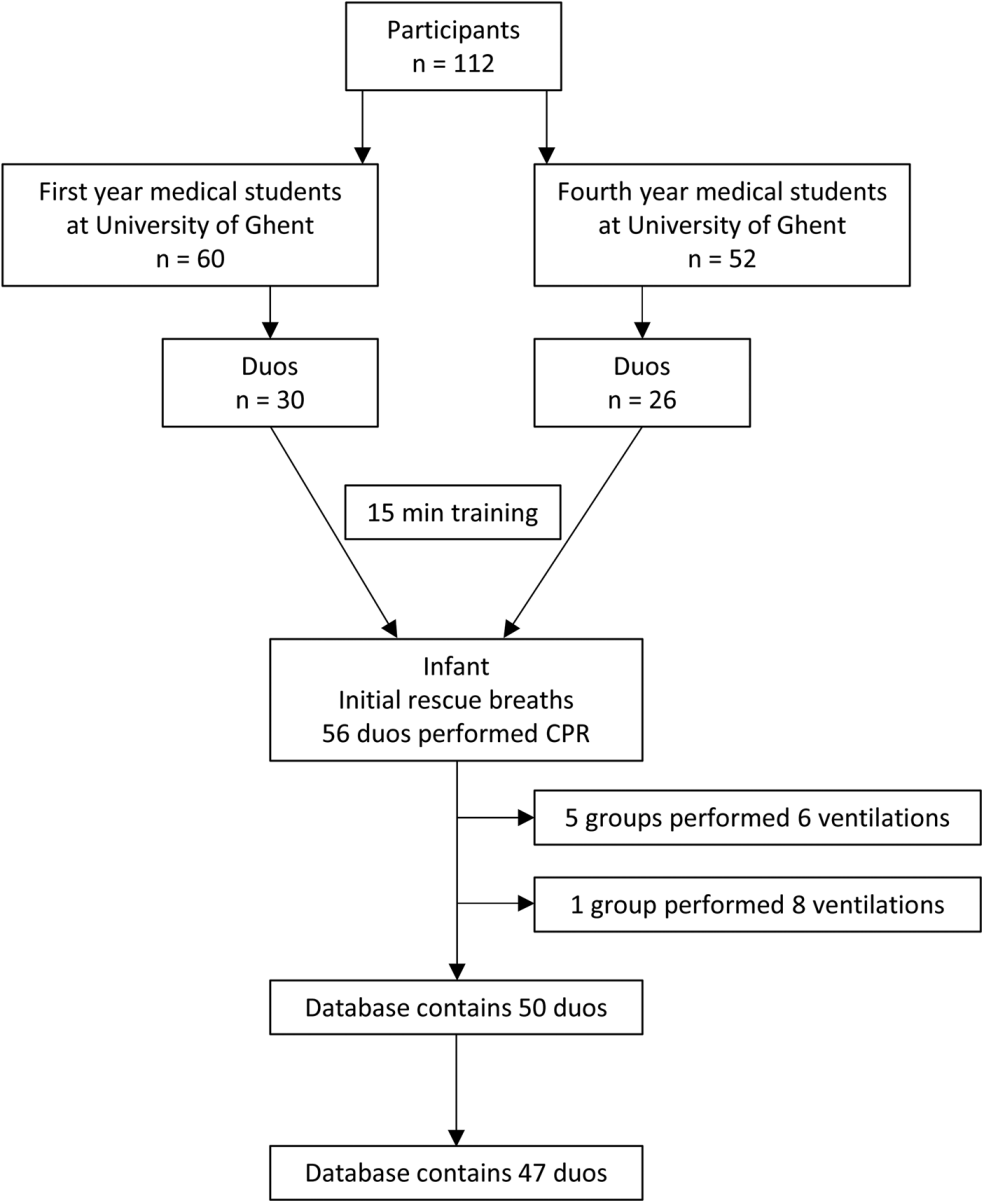
Flow of the participants in the study and during data cleaning.

All participants received a 15 min just-in-time training about the full CPR-cycle including the use of bag-mask for ventilation (Supplementary Material S1). For the use of the BMV, the double C-grip technique was taught where pressure is applied to the hard part of the mask. The importance of a free airway was mentioned. Extra attention was given to the 5 IRB, the 2 thumbs technique used for compressions and the neutral head position (10, 11). After this 5 min introduction course, students had 10 min left where they could practice on manikins with feedback from the instructors. After that, students in duo performed five cycles of CPR with BMV on an infant manikin (Figure 1, Supplementary Figure S1). IRB for infants were performed by a single rescuer (person 1 in Supplementary Figure S1), further ventilations (15:2 sequence) were performed according to the two-person technique (person 1 together with person 2 in Supplementary Figure S1). BMV was administered using a BAG II child resuscitator and a size 1 Disposable mask (Laerdal®, Stavenger, Norway).

Primary outcomes focused on frequency and tidal volume [TV] of IRB ventilations (ml) delivered, secondary outcomes on the mean IRB ventilation volume, on compression depth, recoil, and hands-off time (seconds), all registered using the Resusci Baby QCPR manikin (Laerdal®). The primary outcome consisted of the difference in the % of medical student duos providing at least 2 effective IRB between 2 and 5 attempts. Table 1 includes the numbers allowing calculation of the proportions of success with two and five attempts. We excluded from further analysis those student duos that failed to perform these 5 IRB as part of their BLS algorithm. Due to uncertainty about the amount of TV delivered, we also excluded those duos delivering volumes above 60 ml (maximum volume reported by infant QCPR system). Although the standard normal for TV with the infant QCPR system is set at 20–40 ml, the 40–60 ml interval was also investigated as an alternative acceptable normal, considering that an estimated normal TV according to ERC guidelines would be around 6–8 ml/kg ideal body weight.

**Table 1:** Cross-classification of the number of duos that performed at least two correct ventilations (considering 20–60 ml target volumes) within the five IRB and alternatively only considering the first two of these five IRB.

Success = at least 2 effective IRB	Success with 2 IRB	No success with 2 IRB	
Success with 5 IRB	26	8	34
No success with 5 IRB	0	13	13
	26	21	47

Chi-square testing for categorical and ordinal variables was used in statistical analysis. For continuous variables normality was checked by using the Shapiro-Wilk test and by looking at the QQ plots and for further analysis were either used the unpaired Student's *t*-test or the Mann–Whitney-U and the Wilcoxon Signed-Rank test accordingly (IBM SPSS Statistics 27). The significance level was set at 0.05. The exact 95% confidence interval [95% CI] around a single proportion was calculated using the Clopper-Pearson method and differences between proportions were separately analysed using the Miettinen-Nurminen method (Statsdirect® 3.3.5).

## Results

The 112 participating students were combined in 56 duos, with *n* = 30 duos first year students (mean age: 19 years; 41 women, 19 men) and *n* = 26 duos fourth year students (mean age: 22 years; 39 women, 13 men), all in medicine. Nine duos were not withheld for further analysis due to either incorrect number of IRB (*n* = 6) or (excessive) unmeasurable tidal volumes (*n* = 3), thus finally including 47 duos (Figure 1).

**Figure 2:**
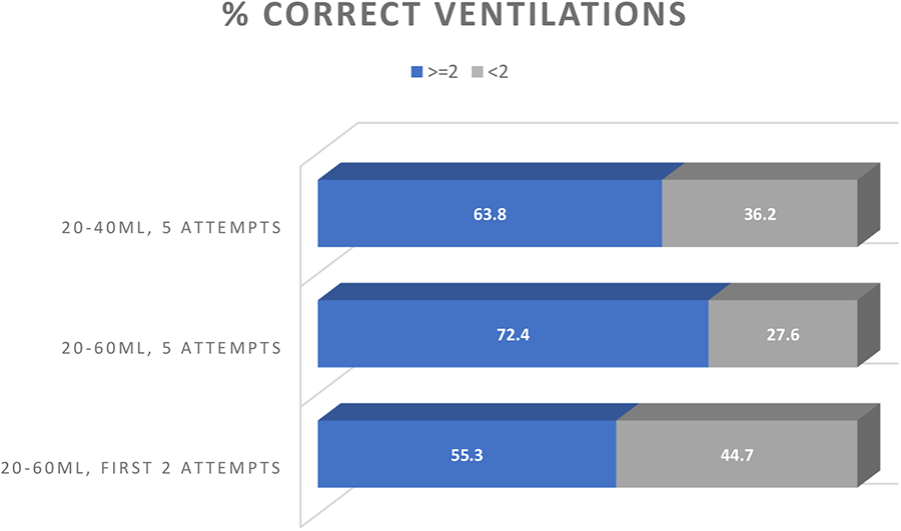
Correct ventilations as part of the IRB. Percentages of duos that performed at least two correct ventilations (considering either 20–4 ml and 20–60 ml target volumes) within the five IRB and alternatively only considering the first two of these five.

The primary outcome consisted of the eventual number of IRB reaching an adequate TV. Considering a target TV of 20–40 ml, 63.8% [95% CI (48.5; 77.3)] were able to perform at least two correct ventilations (Figure 2). If the target was set at 20–60 ml, this increased to 72.4% [95% CI (57.4; 84.4)]. If only the first two ventilations of the set of five would have been taken into account, then 55.3% of the duos [95% CI (40.1; 69.8)] would have succeeded. Therefore, the result for the primary outcome (i.e., the difference in the proportion of 2 effective IRB) was 17.0%, 95% confidence interval [5.4; 28.0].

The secondary outcome consisted of the mean IRB ventilation volume achieving the QCPR normal (20–40 ml). This resulted in 59.6% of the duos reaching successfully this endpoint. Allowing an interval of 20–60 ml increased the number of duos reaching an appropriate mean IRB ventilation volume to 68.1%. However, 31.9% of the duos provided a mean TV below 20 ml. The mean TV delivered was 23.9 ml (±13.2).

## Discussion

The hypothesis was verified in this manikin study that states 5 attempts are strongly recommended in infant CPR to obtain 2 effective IRB. Five attempts may probably compensate for a low level in training, lack of experience and head positioning of the infant. Although the hypothesis was verified, these results should be put into perspective. First of all, there is no uniform definition of “effective” beyond the theoretical normal tidal volume and the clinical “adequate chest rise”. In our study either the predefined QCPR normal (20–40 ml) or a broader TV interval (20–60 ml) were applied, but it should be acknowledged both are still arbitrary. Whilst infant manikins are developed to closely resemble the true infant, they differ from clinical reality (12, 13).

Second, bag-mask was applied to deliver ventilations whereas for infant lay BLS rescuers these are most often performed by mouth over mouth-nose. Choosing for bag-mask was a direct consequence of the COVID pandemic. However, this changed reality brought BMV to the forefront anyhow, not only in paediatric advanced life support but also in BLS for those with sufficient training in its use (1). Our results thus cannot be extrapolated to mouth-to-mouth-nose ventilations, although the available literature suggests this is equally not an easy skill in adults, let al.one in infants, especially if not trained repeatedly (14–17). Moreover, even before the COVID pandemic, there was already a clear reluctance from professional rescuers to perform mouth-to-mouth in adult patients (18, 19). This is likely less an issue for infant BLS, but still relevant (1, 20).

Contrary to AHA guidelines, the ERC 2021 guidelines still advise IRB as part of the paediatric BLS algorithm (1, 21). Presuming that first ventilations might be suboptimal, the authors advise to perform five initial breaths, based on the unproven hypothesis that these would more likely result in at least two effective ones. In our sample, using BMV, only about half of participants were able to deliver two effective rescue breaths with their first two ventilations. Allowing five initial breaths increased the number, reaching two effective ventilations to almost three out of four. This is the first study to our knowledge who explored this hypothesis.

It was chosen to deliberately include only participants with limited to no previous experience with BMV in children, assuming that results would be markedly better in those with paediatric ALS training. All participants received a 15 min just in time training, considering this a low intensity training with effect mostly for easy or already previously acquired skills, be it possibly in different settings. More than 70% of our participant teams were able to perform IRB effectively, suggesting BMV could be opportune to teach to this type of target groups. The 15-minute training prior to the study may be accountable for a bias in the obtained results and will not occur in real life situations, forming an argument in favour of 5 IRB.

One of the problems identified in those participants not able to deliver effective breaths, was an inaccurate not-neutral head position which resulted in a partially blocked airway. Furthermore, some of the teams—biased by previous knowledge and feedback training—were overly aware of the risk of hyperinflation and tended to ventilate with very small volumes. Such fear of doing harm when resuscitating an infant has been described previously (22).

To optimise numbers, we did not strictly randomised between two and five IRB but used the first two IRB as equivalent to a 2 IRB approach. Given that students tried to obtain adequate ventilation with each individual rescue breach, we do not think this induced any significant bias.

Finally, this study did not look at the overall impact of adding (rescue) ventilations to the BLS algorithm, only at the effectiveness of the first five rescue breaths (22). Once started, the actual delay to first compression of five vs. two initial compressions is typically less than 10 s.

## Conclusion

At least half of the participants in our infant manikin study only “needed” their first two rescue breaths with bag-mask to perform two effective ones. However, five IRB allowed a higher proportion of them to reach this goal. Considering the limited delay to compressions and the importance of uniformity in the paediatric BLS algoritm, no sufficient argument was found to change the five IRB, even for those competent in bag-mask ventilation. To conclude, these findings support current ERC recommendations to start with 5 IRB.

## Data Availability

The raw data supporting the conclusions of this article will be made available by the authors, without undue reservation.
