# Supplementary material for: Five vs. two initial rescue breaths during infant basic life support: A manikin study using bag-mask-ventilation
Source: Front Pediatr. 2022 Dec 7;10:1067971. doi: 10.3389/fped.2022.1067971 (PMC9792851; doi:10.3389/fped.2022.1067971)
Supplement: Supplementary file 1 [file Datasheet1.pdf]

Supplementary Material 1:

First, we are going to give a demonstration of the BLS cycle for an infant. You will then have 5 minutes with the manikin to practice in groups of 2. In the meantime, we will correct any mistakes we see. You can choose who takes on which position. When the practice is over, you will move on to the test section. You're going to perform 5 cycles of BLS on each manikin. Your actions will be recorded.

Demonstration infant

Person 2: Hello, baby, can you hear me?

I listen to the breathing for a maximum of 10 seconds, while simultaneously looking at whether the chest rises or not, and feeling if they are breathing against my jaw.

...

No response, I'm going to start with 5 initial rescue breaths.

Make sure to keep the head neutral. Since an infant's ventilation bag is smaller than an adult's, you only need to squeeze the balloon minimally.

When the 5 initial rescue breaths are given, I switch to a double grip.

I place both thumbs on the mask and my palms rest on the forehead.

Person 1: After the first 5 initial rescue breaths I'll immediately start with 15 compressions.

I circle the torso with both hands and at the lower part of the sternum, I give 15 compressions with my 2 thumbs.

Afterwards, I give 2 ventilations with the self-inflating bag.

Person 2: This cycle of 15 compressions followed by 2 ventilations is performed 5 times. But be aware that you do not forget to give those 5 initial rescue breaths first.

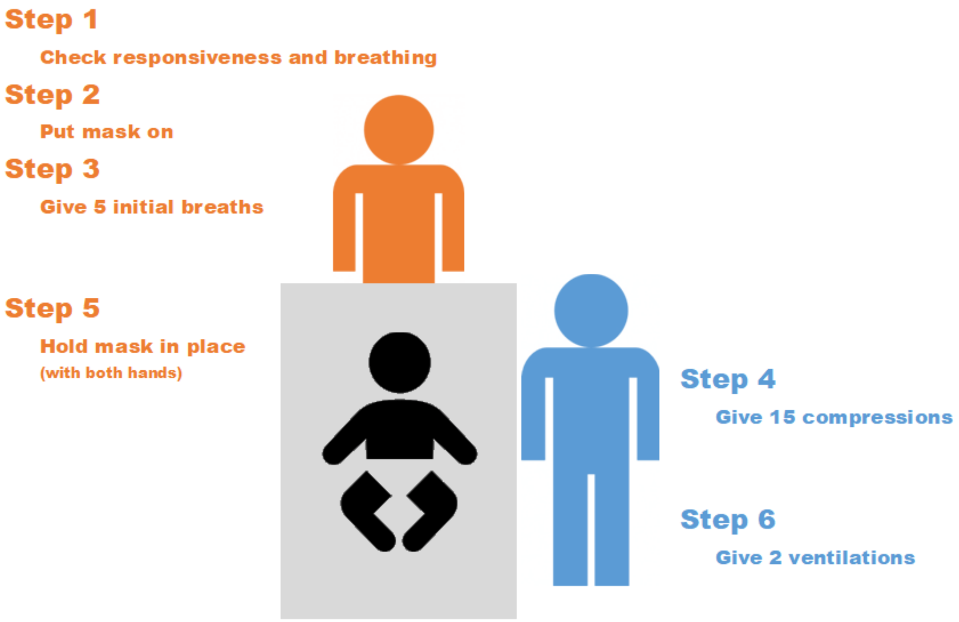

Supplementary Figure 1: Basic life support protocol used for infant CPR during the study. Person 1 is represented in orange and person 2 in blue. Person 1 checks responsiveness and breathing and starts the 5 initial rescue breaths. Afterwards, he places both hands on the mask. Person 2 starts compressions and gives the following ventilations during 5 cycles. During the just-in-time training, participants could practice both position (person 1 and person 2). After the introduction course, the participants were sent to the back of the room where each group performed CPR for 5 cycles, on the infant manikin. The same type of manikins were used for practice and registration by the SimPad.
